# Pediatric Anaphylaxis: A 20-Year Retrospective Analysis

**DOI:** 10.3390/jcm11185285

**Published:** 2022-09-07

**Authors:** Maria De Filippo, Martina Votto, Maria Albini, Riccardo Castagnoli, Mara De Amici, Alessia Marseglia, Alessandro Pizzo, Gian Luigi Marseglia, Amelia Licari

**Affiliations:** 1 Pediatric Unit, Department of Clinical, Surgical, Diagnostic and Pediatric Sciences, University of Pavia, 27100 Pavia, Italy; 2 Pediatric Clinic, Fondazione IRCCS Policlinico San Matteo, 27100 Pavia, Italy; 3 Laboratory of Immuno-Allergology of Clinical Chemistry, Fondazione IRCCS Policlinico San Matteo, 27100 Pavia, Italy

**Keywords:** adolescents, allergic diseases, anaphylaxis, children, intramuscular adrenaline

## Abstract

Background. Anaphylaxis is a steadily increasing global problem defined as an acute hypersensitivity multisystem reaction that is potentially fatal. In the pediatric age, the leading cause is food. In other allergic diseases, intrinsic heterogeneity has been reported in the clinical presentation, severity, and triggers of anaphylaxis. This study analyzes the features and management approach of the anaphylactic reactions in children evaluated at the pediatric clinic in Pavia. Materials and methods. A retrospective study was conducted on patients with anaphylaxis between 2001 and 2021. Results. A total of 148 patients with a median age of 5 years were enrolled, and 80% of the patients had other atopic comorbidities that were correlated with the severity of anaphylaxis. The main trigger of anaphylaxis was food. Most reactions involved mucocutaneous, respiratory, and gastrointestinal systems, and occurred at home. Adrenaline was administered only in a minority of cases. Conclusions. Considering that anaphylaxis is a potentially life-threatening condition requiring prompt management, the use of adrenaline should be implemented. Our data also suggest the importance of educating and spreading awareness of anaphylactic management within the medical community.

## 1. Introduction

Anaphylaxis is a severe systemic hypersensitivity reaction that is usually rapid in onset and may cause death [1]. There are different definitions of anaphylaxis due to its heterogeneity, both from a clinical and pathogenetic point of view [1,2,3,4]. This complexity translates into objective problems in correctly describing anaphylaxis, with repercussions on the study of epidemiology, diagnosis, and treatment of this potentially lethal disease. What is evident from all definitions is that they are intentionally generic in describing the underlying mechanism, clinical presentation, and severity. Anaphylaxis has a very complex yet not fully understood pathophysiology, where many different triggers can activate several biological pathways, leading to different clinical presentations of varying severity. The potential life-threatening nature of anaphylaxis must be stressed because symptom onset and progression are usually rapid, but if addressed immediately and properly treated, the prognosis is generally reasonable.

Anaphylaxis is still considered to be an underdiagnosed, under-reported, and undertreated condition [5]. It appears that anaphylaxis is more frequent than what was previously thought, and its incidence is increasing, especially in young people, but estimating the true epidemiology remains challenging for different reasons [6]. Underdiagnosis is particularly likely during the patient’s first episode, especially if the clinical presentation is mild and/or transient, and if the patient cannot describe their symptoms (e.g., infants) [6].

The absence of mucocutaneous manifestation, which occurs in 10–20% of cases, can also lead to the unrecognition of anaphylaxis [6]. Under-reporting and miscoding are additional issues where the intrinsic multisymptomatic nature of the condition and the current coding systems play a role. Recently, anaphylaxis was introduced in the new classification of allergic and hypersensitivity disorders in the International Classification of Diseases codes 11 (ICD-11), allowing for the collection of more accurate and complete future epidemiological data [6].

Although anaphylaxis is underdiagnosed, recent publications found that the incidence of anaphylaxis has increased in the last few decades, particularly for food-induced reactions [7,8]. These data align with the parallel increase in atopic status and food allergies [7]. The incidence is estimated to be between 50 and 112 episodes per 100,000 person-years, with significant variability among countries and studies [7]. The estimated lifetime prevalence is between 0.3 and 5.1% [7]. Some studies show that the highest incidence rates are recorded during the first years of life, especially the first two years, almost three times higher in children aged 0–4 years than that in other age groups [7,9]. Individuals with asthma are at higher risk of anaphylaxis, and those with severe asthma have the highest risk of all [7,9]. Anaphylaxis is more common in boys until age 10–15, whereas from 15 years onward, the incidence tends to be higher in girls [7,9]. Although anaphylaxis is considered to be a potentially life-threatening condition, the fatality rate is stable compared to the overall increased incidence of anaphylaxis in children, presumably due to the improved recognition of the condition and the publication of international guidelines. Mortality is estimated to be 0.05–0.51 per million people/year for drugs, 0.03–0.32 per million people/year for food, and 0.09–0.12 per million people/year for insect venom [1]. Other important factors associated with increased morbidity and mortality are upright posture and the delayed use of intramuscular adrenaline, whose underuse is widely reported in the literature [10,11]. Unfortunately, this medication is not available in many countries and is widely underused when available, primarily upon recurrence and even when the patient has an adrenaline autoinjector.

Generally, the most common triggers for anaphylaxis are foods, medications, and insect venom. Approximately 20% of anaphylaxis is idiopathic, meaning no trigger is identified. In children and up to the second decade of life, food is the major elicitor, specifically hen eggs, cow’s milk, and nuts, whereas drug- and insect venom-induced anaphylaxis is more prevalent in adults [1,3,7,12]. Moreover, drug- and venom-induced anaphylaxis in children is generally less severe than that in adults [13]. In pediatrics, the most frequent triggers of anaphylactic change according to age and are represented by food, especially milk and egg, followed by dried fruit, fish, and shellfish. Although few epidemiological studies are available on pediatric anaphylaxis, a recent Italian study reports an increasing trend in the last ten years in Italy relating to hospitalization for foodborne anaphylaxis, especially under four years of age [14].

Several aspects of pediatric anaphylaxis are still uninvestigated, such as the lack of clear epidemiological data, the clinical heterogeneity characterization, and the reason of the widespread poor use of intramuscular adrenaline. Although several international observational studies were published in the last few years [15,16,17,18,19,20,21,22,23,24], a few Italian studies assessed pediatric anaphylaxis in the outpatient setting [25,26]. Furthermore, these studies mainly described cases of anaphylaxis in children and adolescents assessed in the emergency department. Given these considerations, the primary purpose of this retrospective study is to describe the clinical features of anaphylaxis in pediatric patients followed in the outpatient clinic, revising the causes, and analyzing the therapeutical adherence to guidelines. Specifically, this study also assesses the following points in children with anaphylaxis: (i) the demographic characteristics, (ii) the main triggers, (iii) the presence of risk factors that may predispose to anaphylaxis and a more severe reaction, (iv) the characteristics of the reactions, and (v) management approach.

## 2. Materials and Methods

### 2.1. Study Design

A retrospective study was conducted on pediatric patients with anaphylaxis followed at the Immunology and Allergy Outpatient Unit of the Pediatric Department at San Matteo Hospital in Pavia between April 2001 and April 2021.

### 2.2. Patients and Data Collection

We included pediatric patients (0–18 years) who had received a clinical diagnosis of anaphylaxis according to the anaphylactic guidelines from the World Allergy Organization and European Academy of Allergy and Clinical Immunology (WAO/EAACI) [1,2,3]. All patients underwent diagnostic tests (skin and/or in vitro tests) to investigate the culprit trigger of anaphylaxis and/or oral challenge to confirm the allergy. Skin-prick tests and/or prick-by-prick tests were considered to be positive when the mean wheal diameter was ≥3 mm, while specific IgE if ≥0.35 kU/L. Molecular diagnostics were performed through the UniCAP method (Thermo Fisher Scientific, Uppsala, Sweden). All enrolled patients received an adrenaline autoinjector with an emergency action plan.

Exclusion criteria included the absence of clinical anaphylactic criteria, and missing data about the reaction or the results of the diagnostic and therapeutic workup.

Medical records of included patients were reviewed in order to collect data on the following parameters:

1. Demographics: date of birth, age at diagnosis (infant younger than 6 months, preschooler under 6 years old, schooler age from 6 to 12 years old, adolescent older than 12 years old), gender, ethnicity.
2. Comorbidities: allergic asthma diagnosed according to the GINA guidelines [27], allergic rhinitis diagnosed according to the ARIA guidelines [28], atopic dermatitis diagnosed according to the Italian guidelines [29], and eosinophilic gastrointestinal disorders diagnosed according to the international guidelines [30].
3. Anaphylactic reaction: date, location, cause, cofactors (infections, NSAIDS, physical exercise, alcohol), clinical manifestations (cutaneous, gastrointestinal, respiratory, cardiovascular, neurological), time lapse between exposure to allergens and onset of symptoms, and severity grading according to Sampson. This system comprises five levels of anaphylaxis: cutaneous, gastrointestinal tract, respiratory, cardiovascular, and neurological involvement [31]. In our study, Levels 1 and 2 correspond to mild anaphylaxis, Level 3 to moderate anaphylaxis, and Levels 4 and 5 to severe anaphylaxis.
4. Treatments of anaphylaxis.

All data were extracted from electronic medical records (Fenix™, EL.CO. S.r.l. Savona, Italy software). Every patient identifier (name and surname) was replaced with a specific numeric code. Data were collected and managed in compliance with European Union General Data Protection Regulation (GDPR). All patients provided written informed consent. The Ethical Committee approved this study (protocol number 0003241/22).

### 2.3. Statistical Analysis

Results are presented as absolute and relative frequencies. Subgroups were compared using a chi-squared test. The statistical significance for all analyses was defined as a *p*-value < 0.05. The statistical analyses were performed through GraphPad Prism version 9.3.0 (San Diego, CA, USA).

## 3. Results

A total of 148 patients with anaphylaxis were enrolled during the study period. The mean age at the anaphylactic episode was 6.1 ± 4.8 years, with a median of 5 years (from 1 month to 18 years of age). At the moment of the reaction, 7% of patients were younger than 6 months, 48% were younger than 6 years, 30% were between 6 and 12 years, and 15% were older than 12 years (Table 1). Most patients were males (61%) among all age groups.

Most patients (83%) had a history of atopic comorbidities: 60% allergic rhinitis, 46% atopic dermatitis, and 39% allergic asthma. In addition, 4% had a diagnosis of mastocytosis, and 2% of eosinophilic esophagitis. Allergic rhinitis was the most common comorbidity among all age groups. The proportion of patients with allergic rhinitis was 73% in adolescents, 61% in school-age children, and 65% in preschoolers. The proportion of patients with asthma was 55% in adolescents, 43% in school-age children, and 37% in preschoolers. On the other hand, atopic dermatitis was the only comorbidity found in children younger than 6 months.

Most reactions (66%) occurred at home, whereas only 5% occurred at school. All characteristics of patients are summarized in Table 1.

The presence of comorbidities is a risk factor for more severe anaphylactic reactions. Table 2 shows the frequency of mild reactions (severity grade ≤ 2) and moderate to severe reactions (severity grade > 2) according to comorbidities. Asthma, mainly if not controlled, is a significant risk factor for severe anaphylaxis (*p* 0.04).

In 123 patients, anaphylaxis was induced by foods. In children younger than 6 months, foods were the only identified triggers. Other causes included venom by insect stings and exercise-induced anaphylaxis. One patient had anaphylaxis due to a drug, and one patient to latex during sublingual allergen-specific immunotherapy. Five patients had idiopathic anaphylaxis. Foods implicated in food-induced anaphylaxis were tree nuts (30%), milk (19%), eggs (15%), peanuts (12%), fish and crustaceans (7%), fresh fruits (7%), and seeds (6%); single patients developed anaphylaxis to wheat, soy, garlic, and spices. Data are summarized in Table 3.

Milk was a particularly relevant trigger in children younger than 6 months, where it accounted for 81% of cases of food-induced anaphylaxis, whereas it was responsible for 17% of cases in children aged younger than 6 years old, 6% of cases in children aged 6 to 12 years, and no cases in children older than 12 years. Tree nuts were a relevant trigger in children younger than 6 years old, accounting for 32% of cases, and children aged 6–12 years, accounting for 38%. In adolescents, tree nuts were responsible for only 14% of cases.

Insect-sting anaphylaxis occurred in 14 patients, and the implicated Hymenoptera were wasps in 64.3% of cases, bees in 28.6% of cases, and a hornet in only one case. Exercise-induced anaphylaxis occurred in 4 male patients. The only case of anaphylaxis to a drug was in a 2-year-old boy who had received ceftriaxone due to pneumonia. Latex-induced anaphylaxis occurred in a 17-year-old boy with a known allergy to latex during sublingual immunotherapy at the third administration after three drops of a 500 mcg/mL solution.

In 5 cases, it was impossible to identify the trigger of anaphylaxis either because the reaction had developed after no apparent trigger or after eating meals with different foods to which the patient had not been sensitized.

The presence of cofactors such as physical exercise, infections, use of NSAIDs, or alcohol was evaluated: physical exercise was identified as a cofactor in four reactions.

In most patients, anaphylaxis is mainly a combination of mucocutaneous, respiratory, and/or gastrointestinal symptoms. The following clinical manifestations were present in the studied patients: mucocutaneous (99%), respiratory (65%), gastrointestinal (59%), glottis edema (15%), neurological (14%), cardiovascular (12%), and loss of consciousness (3%). Results are summarized in Table 4.

Gastrointestinal symptoms appeared more frequently in infant children than they did in adolescents (91% vs. 41%). Moreover, school-age children and adolescents had statistically significantly different respiratory tract involvement and glottis edema compared to children younger than 6 years (*p* 0.03 and *p* 0.02, respectively).

All anaphylactic reactions were immediate (within 2 h) following exposure to the trigger. It was possible to gather information about the exact time lapse between exposure and onset of symptoms in only 76 patients (51%), and the mean time was 23 ± 33 min, with a minimum of 1 min and a maximum of 120 min. The median time lapse was 10 min. A biphasic reaction was observed in only four patients.

The list of drugs used to treat anaphylaxis is listed in Table 5. Of all patients, only 18% (*n* = 27) were treated with intramuscular adrenaline: 4% of patients with severity Grades 1–2, 11% with Grade 3, 81% with Grade 4, and 4% with Grade 5.

## 4. Discussion

In our study, we retrospectively collected the most important details of 148 anaphylactic reactions in children and adolescents followed at the Immunology and Allergy Outpatient Unit of the Pediatric Department at San Matteo Hospital in Pavia over the last 20 years. These details delineated the clinical features of Italian children diagnosed with anaphylaxis in a third-level university hospital. In this setting, we attempted to clarify the differences in the clinical presentations of anaphylaxis triggered by different agents and at different ages. Indeed, we described the management of a cohort of children with anaphylaxis and demonstrated that intramuscular adrenaline is still underutilized today. Several studies focused on the literature on food allergies (e.g., nut and cow’s milk allergies), drug hypersensitivity, or Hymenoptera venom allergy, but there are few studies focused only on systemic reactions due to all these agents in the Italian pediatric population.

As reported in other published studies, most anaphylactic reactions occurred in younger children (55%), with a male preponderance in all age groups [32,33,34]. Atopic diseases were present in 80% of the patients in our sample, with asthma and allergic rhinitis diagnosed primarily in school-age children and adolescents, and atopic dermatitis prevailing in infants and preschoolers, confirming what was observed in the European Anaphylaxis Registry [35]. Other less common comorbidities such as mastocytosis or eosinophilic esophagitis were also present in our cohort. According to the literature, comorbidities, especially severe or uncontrolled asthma, predispose to anaphylaxis and more severe reactions. The proportion of moderate-to-severe anaphylactic reactions was higher in asthmatic patients than that in nonasthmatic ones, and patients with uncontrolled asthma compared to patients with controlled asthma.

Several studies reported that most anaphylactic reactions occur in private homes and outdoor locations, followed by hospitals, schools, and restaurants [35,36]. Our study had similar results, with 66% of reactions occurring at home. This emphasizes the importance of the awareness of anaphylaxis and symptom recognition by healthcare professionals, patients’ caregivers, childcare professionals, and food services, especially considering that most anaphylaxes are food-induced.

In our sample, according to the European Anaphylaxis Registry, foods were the leading cause of anaphylaxis, followed by insect stings. In children younger than 6 months, food was the only elicitor for anaphylaxis, dominated by cow’s milk, as reported by most studies [37,38]. Tree nuts were relevant elicitors at all pediatric ages. In our study, walnuts and hazelnuts were the most implicated tree nuts. These are the most allergenic tree nuts in the United States and Europe [39]. There were no cases of anaphylaxis to cashew nuts in our sample, diverging from other studies, where this tree nut was also a frequent elicitor [36]. Eating habits could explain this difference among countries. After tree nuts, milk, and eggs, the peanut was our series’ fourth most common trigger of food-induced anaphylaxis and the second most cause of food-induced anaphylaxis in school-age children. In adolescents, fish was the most prevalent elicitor, and all three reactions were caused by shrimps, unlike in preschoolers, where various types of fish were involved.

Anaphylaxis to insect stings occurs in less than 1% of children, and the severity of the reactions is generally milder than that in adults. There is a 10% chance of a subsequent similar or milder reaction in children with cutaneous systemic reactions, but only a 1–3% chance of a more severe one. The risk of anaphylaxis increases with multiple stings at one time or repeated stings in a short interval (e.g., in the same summer). Moreover, initial reaction severity is linked to the risk of recurrence: the more severe it is, the higher the risk. The risk generally declines when a person is not stung for a long time, but it remains in the range of 20–30% even after 10 years.

On the other hand, very frequent stings (more than 200 annual stings, as in beekeepers) tend to induce tolerance. This observation is in line with the high efficacy of venom immunotherapy, which is particularly effective and long-lasting in children. The onset of symptoms is generally within 10 to 30 min of the sting, and the slower the onset is, the less the chance of life-threatening anaphylaxis is. A rapid onset reaction to stings may be related to an underlying mast-cell disorder that should be investigated. Insect stings are the most common cause of anaphylaxis in patients with indolent systemic mastocytosis, which is associated with severe venom-induced anaphylaxis. Elevated levels of tryptase, even in those patients without a diagnosis of mastocytosis, are also linked to very severe reactions to stings. The major culprit insects of anaphylaxis belong to the order of Hymenoptera, which includes bees (honeybees, bumblebees), vespids (yellow jackets, hornets, wasps), and stinging ants. Cross-reactivity between the venoms of different insects can occur, particularly among vespids, and between honeybees and yellow jackets. Interestingly, a patient can react to one sting and not another, even from the same species, but this could be explained by the fact that the amount of venom injected in a sting can vary conspicuously, leading to the false impression that the patient is no longer allergic [40].

Exercise-induced anaphylaxis occurred in 3% of patients. Among these, it was possible to diagnose food-dependent exercise-induced anaphylaxis in only one patient, and the culprit food was an apple. Drugs and latex were responsible for one case each. The low frequency of drug-induced anaphylaxis reported in our sample is probably due to selection bias, since our outpatient unit is not specialized in drug allergy. The main drug triggers of anaphylaxis in children are antibiotics, especially ß-lactams, and nonsteroidal anti-inflammatory drugs (NSAIDs) [7]. Other less common triggers are chemotherapeutic agents, monoclonal antibodies, small molecules, drug contaminants, and perioperative medications [4]. Latex-induced anaphylaxis occurred in a 17-year-old boy with a known severe allergy to latex during sublingual immunotherapy at the third administration. The low prevalence of anaphylaxis during AIT confirms the excellent safety profile of AIT in the pediatric population, according to a European survey [41,42].

A recent systematic review reported that approximately 10% of pediatric anaphylaxes are classified as idiopathic after an extensive evaluation [43]. In a 9-year retrospective study, Silva et al. [34] reported a 7% frequency, while Hoffer et al. found a 5% frequency in a 12-year retrospective study [23]. In our sample, it was impossible to reach etiological diagnosis in 3% of children. In these cases, in vivo and in vitro tests failed to identify the culprit food.

The high rate of etiological confirmation and epinephrine autoinjectors (EAIs) prescription at our allergy outpatient unit confirms the importance of patients’ referral to an allergy specialist, as indicated by most guidelines [1,2,3]. Moreover, the investigation of molecular components confirmed that most patients were sensitized to allergenic molecules associated with severe systemic reactions [44]. Studying these allergenic molecules constitutes an additional tool that may help physicians in assessing the likelihood of clinical reactivity to certain foods, especially when skin-prick tests and food-specific IgE levels are below the published decision points, as what occurred in a proportion of our patients.

According to the literature, mucocutaneous symptoms are the most common manifestations, followed by respiratory and gastrointestinal symptoms [35,36]. Respiratory involvement was more common in older children and adolescents than that in preschoolers, and we did not observe a significant difference in respiratory symptoms between asthmatic and nonasthmatic patients. Gastrointestinal involvement was relevant in all age groups, and particularly children younger than 6 months. These findings were also reported in previous studies, reinforcing the importance of including gastrointestinal symptoms in the diagnostic criteria of anaphylaxis, especially at younger ages [32,45]. Most reactions developed within 30 min from exposure to the trigger and were mainly moderate to severe (Sampson’s severity grades 3–5), often occurring away from healthcare settings, highlighting the importance of rapid recognition of symptoms and prompt treatment, especially considering the unpredictable course of anaphylactic symptoms.

Despite the limited small number of enrolled patients, this study reported that only 18% of children and adolescents with anaphylaxis were treated with intramuscular adrenaline, highlighting a concerning underuse of this pivotal therapy. Although first-line treatment with intramuscular adrenaline is reinforced in all international anaphylactic guidelines, many studies observed that it is still widely underused, with rates ranging from 25 to 33% of cases treated in the emergency department [32,33,34]. There are several reasons for not using intramuscular adrenaline, such as the failure to identify anaphylactic symptoms, the underestimation of initial reaction severity, hesitancy and the spontaneous resolution of symptoms, reliance on oral antihistamine, concerns about the safety of adrenaline, fear of the injection, and also not carrying the autoinjector all the time [46,47]. Moreover, IM adrenaline became the first-line treatment of anaphylaxis in 2014 [3], 13 years after the study period. Lastly, EAIs are often underused by patients and caregivers during anaphylactic recurrence [46,48]. According to several reviews, the most administered medications were antihistamines and corticosteroids, considered to be additional interventions in current guidelines [1,2,3,4]. Although the spontaneous resolution of anaphylaxis has been reported, every reaction should be promptly treated upon anaphylactic symptom identification, especially considering that the reaction is largely unpredictable and potentially life-threatening, and that the delayed use of adrenaline is correlated with an increased risk of mortality and biphasic reactions [11]. Although we did not report any fatality in our series, and fatal anaphylaxis constitutes less than 1% of the total mortality risk in people with known allergies, this low risk exists [49]. Biphasic reactions are estimated to occur in 15–20% of cases, occurring up to 72 h after resolution of initial symptoms, but more commonly within 12 h. For this reason, guidelines emphasize the need to observe patients for at least 6–24 h, especially those experiencing severe anaphylaxis and requiring multiple doses of adrenaline [1,2,3,4]. In our study, the biphasic reaction occurred in only four patients from 3 to 24 h after initial symptom cessation.

At the time of discharge from a healthcare setting, it is crucial to inform patients and their caregivers about the risk of anaphylactic recurrence, which is estimated to range from 26.5 to 54% during a follow-up time of 1.5–25 years [7]. Guidelines suggest that patients receive at least one adrenaline autoinjector or a prescription, with clear instructions on when and how to use it. Moreover, patients should be referred to an allergist for confirmation of the suspected trigger to better tailor the long-term management of anaphylaxis, which is based on trigger avoidance, allergen immunotherapy if indicated, and the control of concomitant diseases [1,2,3,4,50]. Thus, providing adequate training to all patients at risk of anaphylaxis and their caregivers is vital, covering symptom recognition, when and how to administer self-injectable adrenaline, and reinforcing the importance of continuously carrying the EAI. Training should be especially effective and based on a multidimensional approach, a written action plan, and periodic medical re-education [46]. A recent study showed that, despite most patients or caregivers receiving training and responding correctly to a survey regarding EAIs, they rarely used the autoinjectors when needed. Only 36.7% felt confident about using the EAI after training, 42.6% felt anxious, and 15.4% felt fear [51]. Therefore, psychological factors should be considered and addressed, too, as they can negatively impact autoinjector use. Physiological intervention could also be helpful, considering that the life of patients with a history of anaphylaxis and one of their caregivers is negatively impacted, especially in the case of food allergy [41]. Indeed, the constant effort to strictly avoid culprit allergens, the uncertainty of possible contaminations, and the fear of being unable to manage a reaction are all sources of anxiety. Training should be necessary for people in contact with children at risk of anaphylaxis, such as professionals within healthcare, education, and childcare, as preparedness is often suboptimal [46]. Several studies [11,46,47,48,49,50,51,52] underlined the primary concern of the lack of knowledge and misconception about the management of anaphylaxis among the medical community, emphasizing the need to spread awareness, starting from medical universities.

## 5. Conclusions

In conclusion, this study confirmed that anaphylactic triggers varied with age. The more significant causes of anaphylaxis were foods, particularly tree nuts, milk, and eggs, the last two being particularly relevant in children younger than six years of age. The most common clinical manifestations included mucocutaneous and respiratory symptoms in older children, while mucocutaneous and gastrointestinal symptoms, although relevant among all age groups, prevailed in children younger than 6 months of age. According to comorbidities, uncontrolled asthma was a well-assessed risk factor for severe anaphylaxis in our cohort, emphasizing the importance of prompt and immediate management with intramuscular adrenalin and a good asthma control in these patients. In our population, a concerning underuse of adrenaline was reported, suggesting the need to educate clinicians on this life-saving medication and to generally spread awareness on anaphylactic management within the medical community. A referral to an allergist and the regular follow-up visits are crucial to confirm the trigger of anaphylaxis and assess the risk of future recurrence, aiming to reduce this risk through management of comorbidities often present in patients with a history of anaphylaxis. Efforts to educate patients about allergen avoidance strategies, symptom recognition, and the importance of adrenaline autoinjector use should continue, as adrenaline self-injection in the community setting is still suboptimal despite being the first step in anaphylactic management.

## Figures and Tables

**Table 1:** Features of pediatric patients with anaphylaxis.

	≤6 Months	≤6 Years	6–12 Years	>12 Years	Total
	*n* = 11 (7%)	*n* = 71 (48%)	*n* = 44 (30%)	*n* = 22 (15%)	*n* = 148 (100%)
Male sex, *n* (%)	6 (4)	41 (28)	29 (66)	15 (68)	91 (61)
Comorbidities					
Allergic rhinitis, *n* (%)	-	46 (65)	27 (61)	16 (73)	89 (60)
Atopic dermatitis, *n* (%)	4 (36)	36 (51)	19 (43)	9 (41)	68 (46)
Asthma, *n* (%)	-	26 (37)	19 (43)	12 (54.5)	57 (39)
Mastocytosis, *n* (%)	-	3 (4)	2 (4.5)	1 (4.5)	6 (4)
EoE, *n* (%)	-	3 (4)	-	-	3 (2)
Site of anaphylaxis					
Home, *n* (%)	10 (91)	54 (76)	25 (57)	8 (36)	97 (66)
Outdoor place, *n* (%) °	-	4 (6)	9 (20)	7 (32)	20 (14)
Hospital setting, *n* (%) °°	1 (9)	9 (13)	6 (14)	2 (9)	18 (12)
Restaurant, *n* (%)	-	1 (1)	2 (5)	5 (23)	8 (5)
School, *n* (%)	-	3 (4)	2 (5)	-	5 (3)

EoE, eosinophilic esophagitis; ° parks, public gardens, and squares; °° 14 cases occurred during the OFC, one during the latex allergen-AIT, 3 cases during hospitalization (one case was drug-induced anaphylaxis, one case at the first administration of cow’s milk, and one idiopathic).

**Table 2:** The severity of anaphylaxis according to comorbidities.

Comorbidities	Grade ≤ 2	Grade > 2	*p* Value
	*n* = 33	*n* = 115	
Asthma, *n* (%)	8 (14)	49 (86)	0.07
Not controlled asthma, *n* (%)	2 (7)	25 (93)	0.04
Atopic dermatitis, *n* (%)	17 (25)	51 (75)	0.50
Mastocytosis, *n* (%)	2 (33)	4 (67)	0.60

**Table 3:** Anaphylactic triggers.

Triggers	≤6 Months	≤6 Years	6–12 Years	>12 Years	Total
Foods	*n* = 11 (9%)	*n* = 66 (54%)	*n* = 32 (26%)	*n* = 14 (11%)	*n* = 123 (100%)
Tree nuts, *n* (%) °	-	23 (32)	12 (38)	2 (14)	37 (30)
Milk, *n* (%)	9 (81)	12 (17)	2 (6)	-	23 (19)
Egg, *n* (%)	-	12 (17)	4 (13)	2 (14)	18 (15)
Peanut, *n* (%)	-	6 (8)	7 (22)	2 (14)	15 (12)
Fish and crustaceans, *n* (%) °°	1 (11)	4 (6)	1 (3)	3 (21)	9 (7)
Fresh fruits, *n* (%) °°°	-	6 (8)	2 (6)	1 (7)	9 (7)
Seeds, *n* (%)	-	3 (4)	3 (9)	2 (14)	8 (6.5)
Wheat, *n* (%)	1 (11)	-	-	-	1 (1)
Soy, *n* (%)	-	-	-	1 (7)	1 (1)
Spices, *n* (%) °°°°	-	-	1 (3)	1 (7)	2 (2)
**Venom**	***n* = 0**	***n* = 4 (29%)**	***n* = 7 (50%)**	***n* = 3 (21%)**	***n* = 14 (100%)**
Wasp, *n* (%)	-	3 (75)	5 (71)	1 (33)	9 (64)
Bee, *n* (%)	-	1 (25)	2 (29)	1 (33)	4 (28.5)
Hornet, *n* (%)	-	-	-	1 (33)	1 (7)
Drug *	***n* = 0**	***n* = 1 (100%)**	***n* = 0**	***n* = 0**	***n* = 1 (100%)**
Latex **	*n* = 0	*n* = 0	*n* = 0	*n* = 1 (100%)	*n* = 1 (100%)
Exercise	*n* = 0	*n* = 0	*n* = 1 (33%)	*n* = 3 (67%)	*n* = 4 (100%)
EIA, *n (%)*	-	-	1 (100)	2 (67)	3 (67)
FDEIA (apple), *n* (%)	-	-	-	1 (33)	1 (33)
**Idiopathic**	***n* = 0**	***n* = 0**	***n* = 1 (20%)**	***n* = 4 (80%)**	***n* = 5 (100%)**

Exercise-induced anaphylaxis (EIA), food-dependent exercise-induced anaphylaxis (FDEIA). ° Walnut, hazelnut, pistachio. °° Salmon, cod, and shrimp. °°° Kiwi, peach, and banana. °°°° Saffron and paprika. * Ceftriaxone. ** Latex-induced anaphylaxis occurred in a 17-year-old boy with a known severe allergy to latex during sublingual immunotherapy at the third administration after three drops of a 500 mcg/mL solution.

**Table 4:** Clinical manifestations.

Symptoms	Overall	≤6 Years		>6 Years		*p* Value
	*n* = 148	*n* = 82		*n* = 66		
		≤6 Months	≤6 Years	6–12 Years	>12 Years	
		*n* = 11	*n* = 71	*n* = 44	*n* = 22	
Mucocutaneous, *n* (%)	147 (99)	10 (91)	71 (100)	44 (100)	22 (100)	>0.99
Respiratory, *n* (%)	96 (65)	4 (36)	43 (61)	30 (68)	19 (86)	0.03
Gastrointestinal, *n* (%)	87 (59)	10 (91)	42 (59)	26 (59)	9 (41)	0.24
Glottis edema, *n* (%)	22 (15)	-	7 (10)	6 (14)	9 (41)	0.02
Neurological, *n* (%)	25 (17)	-	13 (18)	6 (14)	6 (28)	0.82
Cardiovascular, *n* (%)	17 (11)	-	6 (8)	8 (18)	3 (14)	0.12

**Table 5:** Treatment of anaphylaxis according to age.

	≤6 Months	≤6 Years	6–12 Years	>12 Years	Total
	*n* = 11	*n* = 71	*n* = 44	*n* = 22	*n* = 148
IM adrenaline, *n* (%)	1 (9)	12 (17)	9 (20)	5 (23)	27 (18)
Beta-2 agonists, *n* (%)	3 (27)	7 (10)	6 (14)	5 (23)	21 (14)
Nebulized adrenaline, *n* (%)	-	4 (6)	-	5 (23)	9 (6)
Corticosteroids, *n* (%)	7 (64)	52 (73)	33 (75)	18 (82)	110 (74)
Antihistamines, *n* (%)	8 (73)	58 (82)	27 (61)	17 (77)	110 (74)
Oxygen, *n* (%)	-	3 (4)	-	1 (4)	4 (3)
IV fluids, *n* (%)	2 (18)	3 (4)	3 (7)	4 (18)	12 (8)

IM, intramuscular; IV, intravenous.

## Data Availability

Not applicable.
